# The effectiveness of compression garments on RRI prevention in half- and full-marathon runners: Emulation of a RCT using data from an observational study

**DOI:** 10.1371/journal.pone.0362483

**Published:** 2026-11-12

**Authors:** T. P. C. Franke, N. P. A. Zuithoff, F. J. G. Backx, B. M. A. Huisstede

**Affiliations:** 1 Department of Rehabilitation, Physical Therapy Science and Sports, Brain Center, Rudolf Magnus, University Medical Center Utrecht, Utrecht University, Utrecht, The Netherlands; 2 Department of General Practice, Julius Center for Health Sciences and Primary Care, University Medical Center Utrecht, Utrecht University, Utrecht, the Netherlands; University of Tehran, IRAN, ISLAMIC REPUBLIC OF

## Abstract

**Background:**

Runners use compression garments (CGs) to reduce the risk of lower extremity running-related injuries (RRIs). However, studies investigating if using CGs alters the risk of RRIs in runners are scarce.

**Purpose:**

To study the effect of CGs on the occurrence of lower extremity RRIs in half and full marathon runners.

**Study design:**

Observational cohort study

**Method:**

We emulated a hypothetical target trial, an imaginary RCT, using data from the observational SUMMUM-2017 study. Runners who participated in one of the four cohorts of this study during their preparation for the half or full Utrecht Marathon were followed for 16 weeks. Runners stated whether they used CGs at baseline and reported, every 2 weeks, whether they had sustained an injury, using an online questionnaire. Only runners (n = 329) who had not had a RRI at baseline were included in the analysis. The primary outcome, any lower extremity RRI during any 2-week period, was analyzed with a mixed logistic regression model.

**Results:**

No significant effects of CGs on the odds of a lower extremity RRI were identified in half or full marathon runners (odds ratio 1.07 95% CI 0.48–2.40).

**Conclusion:**

Based on our target trial emulation half and full marathon runners, without RRIs at baseline, who used CGs did not have a smaller or greater risk of getting a lower extremity RRI at any point in time during training for a half- or full-marathon event.

## Introduction

Worldwide, millions of adults participate in running [1,2]. Unfortunately, runners are prone to running-related injuries (RRIs), especially of the lower extremity [3,4]. A variety of measures have been used to prevent, mitigate, or cure RRIs [5]. Nowadays, lower extremity compression garments (CGs), specifically compression socks or tubes, are popular and frequently used by runners and especially by half and full marathon runners [6,7]. The CGs are primarily used by endurance athletes to prevent RRIs or to improve post-exercise recovery, performance, and recovery from RRIs, although prevention of RRIs is the most important reason given by runners for using CGs [8]. Alas, this this topic has received far less attention in scientific studies than the effect of CGs on post-exercise recovery and performance enhancement. Several systematic reviews have reported that wearing CGs appears to decrease muscle soreness after a training or match, decrease creatine kinase activity, and speed up recovery [9–12]. CG use is also reported to increase venous blood flow and the clearance of metabolites, but decrease the clearance of blood lactate [12–14]. The precise physiological and biomechanical mechanisms by which CGs stimulate post-exercise recovery are not fully understood [9,10,15].

To date, no randomized controlled trials (RCTs) have investigated the primary or secondary preventive effects of CGs [15]. A cross-over study of the effects of using CGs on stride impact acceleration (i.e., the impact acceleration and magnitude of the stride of a runner) in runners reported that using CGs significantly reduced the stride impact and hypothetically reduced the occurrence of RRIs [16]. However, more studies are needed to investigate whether wearing CGs can reduce the risk of lower extremity RRIs.

The aim of this study was to determine the effect of CGs on the occurrence of lower extremity RRIs in half and full marathon runners. A priori we hypothesized that CGs use would lower the risk of RRIs. Although RCTs are typically used for comparative effectiveness research [17,18], in some cases data from observational studies can be used for the same purposes, usually because RCTs are not possible or feasible. As such, we emulated a hypothetical RCT using a target trial emulation approach to answer our research question [19,20].

## Method

We aimed to use and approximate the target trial emulation methodology as much as possible. Target trial emulations work in three parts. Firstly, the observational cohort study that has been performed is described. Secondly, the hypothetical target trial, and lastly the method to emulate the target trial using the data of the observational study are described. All three steps consist of a total of nine components, (see Table 1) [20–22]. These components are performed consecutively and helped us to approximate the target trial emulation methodology; i.e., to analyze the data in such a way that it emulates the target trial and minimizes bias as much as possible [22,23]. Within the target trial emulation we aim to analyze the use of a self-selected CG.

**Table 1:** Target trial emulation approach: description of the observational study performed and parts and components the hypothetical target trial, and the emulation of the target trial.

Components	Purpose	Part 1: SUMMUM-2017	Part 2: Protocol of the target trial, i.e., the hypothetical RCT	Part 3: Emulation of the target trial
		Protocol of the observational study performed.	The design of the imaginary RCT.	The emulation of the target trial using the data of the observational study including the assumptions necessary to perform the emulation.
1. Aim			To estimate the effect of CGs on the effect of incurring a lower extremity RRI in injured and uninjured half and full marathon runners.	Same as in the hypothetical target trial.
2. Study design	Describes the study design used.	Prospective observational cohort study.	RCT.	Target trial emulation.
3. Eligibility criteria	Describes inclusion criteria applied to the participants.	Runners, with or without RRI at baseline, had to meet the following criteria: 1) aged 18 years or older; 2) registered for the half or full Utrecht Marathon 2017, 3) adequate Dutch language skills.	Same as in the SUMMUM-2017 study except that only runners without a RRI at baseline will be included.	Same as in the hypothetical target trial.
4. Assignment procedures	Describes if and how runners were assigned to a treatment group.	At baseline all runners reported whether or not they use CGs. Runners chose themselves if and when they used their CGs.	Random assignment to either the group of CG users (intervention group) or non-CG users (control group).	Same as in SUMMUM-2017.
5.Treatment strategies	Describes if and how treatment strategies were applied in the observational study, the target trial, and how they were applied in the emulation.	No treatment strategy were actively applied. Runners reported if they used a self-selected lower leg CGs or no CGs at all. All runners followed their own regular running training schedule during the study period.	1. In the intervention group runners use lower leg CGs during training. 2. In the control group runners do not use CGs. Both groups followed their regular running training schedule during the four month study period.	1. The intervention group consists of runners who reported using any self-selected lower-leg CG at baseline. 2. The control group contains runners who reported not using CGs at baseline.
6. Follow-up period	Describes the duration of the follow-up period.	Runners entered the study at either 16 (Cohort 1 4Mo), 12 (cohort 2 3Mo), 8 (cohort 3 2Mo), 4 (cohort 4 1Mo) weeks prior to the event and would be followed up to the half or full Utrecht Marathon.	Runners were followed for 16 weeks during their preparatory period for the Utrecht Marathon up to the running-event or loss-to-follow-up which ever occurred first.	Runners from all cohorts (cohort 1 4Mo, cohort 2 3Mo, cohort 3 2Mo, and cohort 4 1Mo) were all included in the same emulation.
7. Outcome(s)	Describes the outcome measures used and how it is applied in the emulation of the target trial.	The Dutch version of the OSTRC questionnaire and follow up questions were used to determine if the runner had a lower extremity RRI and the location and type of the RRI. Questionnaires were sent to the runners every two weeks to monitor their RRI status.	The Dutch version of the OSTRC questionnaire would be used to determine if runners had a lower extremity RRIs at any 2-week period during follow-up.	Using the data from the SUMMUM-2017, the outcomes of the target trial were used. Lower extremity RRIs included RRIs at the following locations: hip, groin, buttock, upper leg, dorsal side of the upper leg, knee, lower leg, Achilles tendon, ankle, and foot/toe.
8. Causal contrast	Either an intention-to-treat analysis (investigates the effect of randomly assigning runners to either the CG or non-CG users group) or a per-protocol analysis (investigates the effect of CGs using runners who fully adhere to the intervention) could be performed.	CG use was registered at baseline.	Intention-to-treat analysis.	Intention-to-treat analysis.
9. Data analysis	Describes the data analyses techniques used for each protocol	In order to be included in the analysis runners had to have completed the personal characteristics section of the baseline questionnaire. A multivariate linear mixed model regression analyses was performed.	Analyses would be performed with a logistic Generalized Estimating Equation analysis.	A multivariate linear mixed model regression analyses with a random intercept was performed.

**Abbreviations:** CG, compression garments; OSTRC questionnaire, Mo, months; Oslo Sports Trauma Research questionnaire; RCT, randomized controlled trial; RRI, running-related injury; SUMMUM, SUccess Measurement and Monitoring Utrecht Marathon.

### Design of the SUMMUM-2017 study

Data from the prospective observational cohort study, the SUccess Measurement and Monitoring Utrecht Marathon (SUMMUM)-2017 study [24], were used to emulate a RCT. The SUMMUM 2017 study followed runners during their preparation for the 2017 Utrecht (half) marathon. The study was approved by the University Medical Center Utrecht Ethics Committee (protocol number 16/503).

From September 2016 up to the day of Utrecht Marathon (March 19 2017) runners were recruited during registration for the half- or full-marathon, via a newsletter and a public symposium on RRIs. Those interested in participating were sent an information letter. Informed consent was provided before a runner could start filling-in the baseline questionnaire. Runners could enter the study at four moments during preparation for the Utrecht Marathon: 16 weeks (Cohort 4Mo [months]), 12 weeks (Cohort 3Mo), 8 weeks (Cohort 2Mo), 4 weeks (Cohort 1Mo) before the running event. In this way, we created four cohorts. The following inclusion criteria were applied: age 18 years of or older at baseline, registered for the half- or full-Utrecht Marathon 2017, adequate Dutch reading and writing skills.

### Procedures

The baseline questionnaire was sent via e-mail to all participants at the four entry moments for the observational study (Fig 1). Subsequently, the participants were invited to fill-in the follow-up questionnaires regarding their RRI status every two weeks up to the date of the event. Participants were given five days to complete each online questionnaire; reminders were sent every two days to participants who had not started or finished the questionnaires. All questionnaires were sent by using NetQuestionnaires (NETQ Healthcare BV, NETQ Insights BV, Utrecht, The Netherlands).

**Fig 1:**
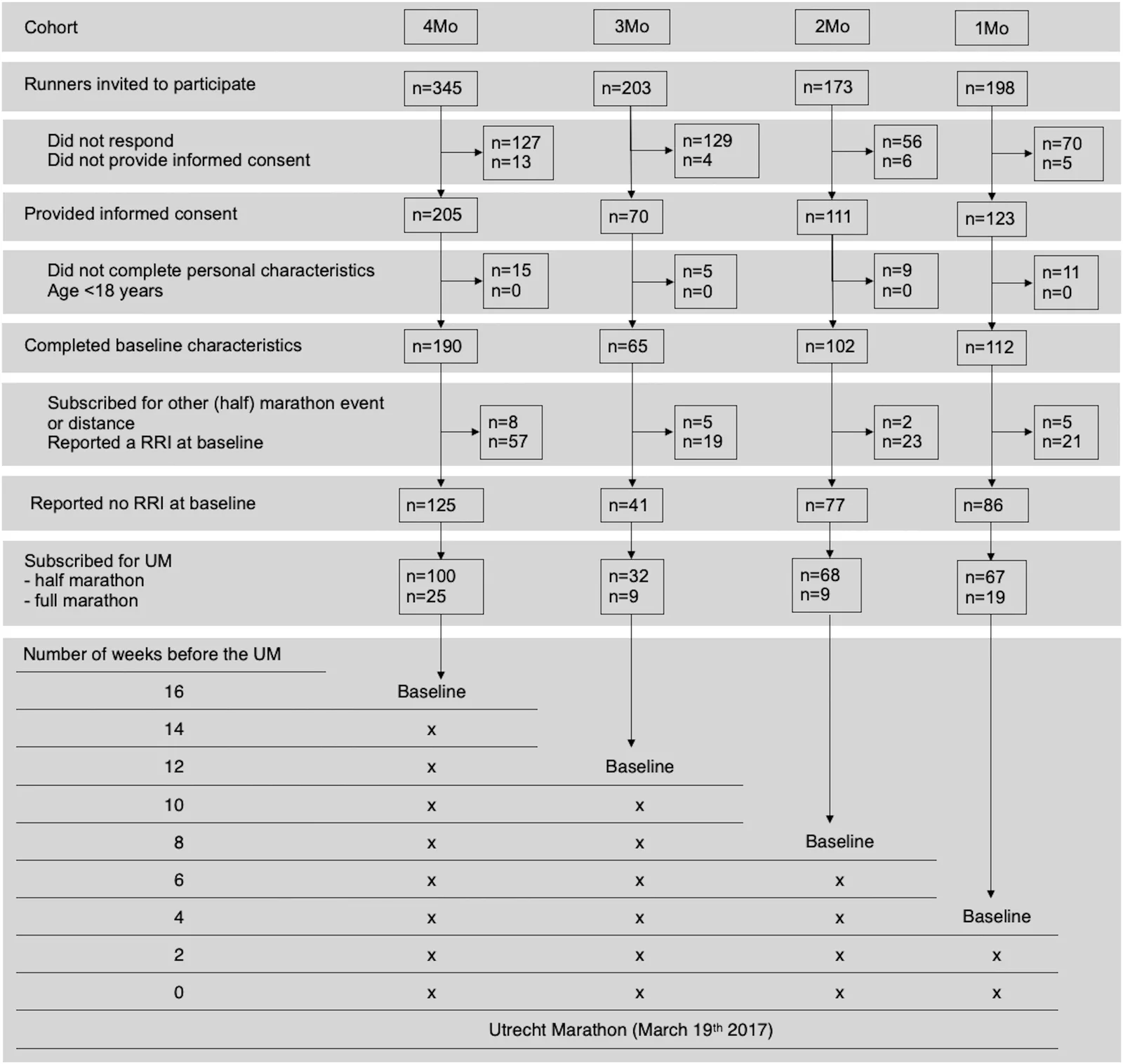
Flowchart of participants. Abbreviations: OSTRC, Oslo Sports Trauma Research Center Questionnaire; Mo, Months; RRI, running-related injury; UM, Utrecht Marathons; x, denotes the bi-weekly follow-up questionnaire.

### Questionnaires

The baseline questionnaire collected demographic information, prior half or full marathon experience, and history of RRIs in the 12-months before runners entered the study. Runners were also asked whether they use CGs. No data on the type of self-selected CG or compliance for using CGs were registered. We used the Dutch version of the OSTRC on health problems to monitor the occurrence of RRIs [4,25]. Details regarding the translation of the OSTRC questionnaire into Dutch are described elsewhere [4]. In brief, the OSTRC enables runners to self-report RRIs, using four multiple-choice questions on sports participation, training volume, and sports performance. By summing the scores of each of the four questions (0–25), the OSTRC severity score (max score 100) was calculated every two weeks.[25]. If the OSTRC severity score was greater than zero, runners were considered injured. Injured runners filled-in additional questions regarding the anatomic location and type of RRI. They were asked whether they had a second or third RRI, and, if so, the OSTRC and the follow-up questions were repeated.

An RRI was defined as any self-reported complaint involving muscles, joints, tendons, and/or bones deemed by the runner to be caused by running. To be classified as a RRI, the complaint [1] had to be present for ≥1 running session, and [2] should have reduced the distance, speed, duration, or frequency of running [4]. Runners were asked not to report blisters or delayed onset muscle soreness.

Before data analysis, all RRIs were evaluated by one researcher in order to determine whether RRIs were reported multiple times. If an individual runner reported multiple RRIs of the same type, at the same location, at different times, either consecutively or non-consecutively, the RRI was considered as one RRI.

### Hypothetical target trial – the imaginary RCT

In this second step we describe the (hypothetical) target trial, the imaginary RCT, for which the objective is to evaluate the effect of CGs on the risk of RRIs.

The objective of the target trial would be to estimate the effect of CGs on the occurrence of lower extremity RRIs in half- and full marathon runners. Ideally, the RCT would include runners in training for a running event such as the Utrecht Marathon. Furthermore, exclusion criteria should be kept to a minimum to obtain an effect of CGs that may be valid for most runners. CG use would be 1:1 randomized, if possible stratified randomization would be considered for runners experience and earlier occurrence of RRIs, to allow for subgroup evaluation in the analysis. Moreover, in the target trial all runners would use the same type of lower leg CGs, with the same pressure gradient, during running. Both CG use and the occurrence of RRIs would be monitored regularly (i.e., every week) to obtain accurate data on compliance and primary outcome. Furthermore, training load would be monitored. The runners would be followed during the final 16 weeks of training before the Utrecht (half) Marathon. Analysis would be performed under intention-to-treat (ITT) principle, additional analysis would focus on obtaining an unbiased estimator that corrects for drop-out and non-compliance.

### Emulation of the target trial using observational data

This section, step 3, describes the components of the emulation of the target trial using data from the observational study, SUMMUM-2017. Component 9 of step 3, i.e., the data analysis, is briefly described in Table 1 and in more detail below. The emulation protocol was defined before the analyses.

In order to mimic the design of the target trial, we included half and full marathon runners from the SUMMUM-2017 study who met all of the target trial eligibility criteria, with one exception. Runners who did not complete the baseline questionnaire and runners with any lower extremity RRIs at baseline were excluded. The above-mentioned four cohorts of the SUMMUM-2017 study were included in the emulation and analyzed as one group.

The outcome measures for the target trial emulation focused on the entire follow-up period for all cohorts. The dichotomous primary outcome, a RRI, was defined as having any lower extremity RRI in any 2-week period during the follow-up period.

In the SUMMUM-2017 study the runners, preparing for the Utrecht (half)marathon, reported at baseline whether they use CGs while they are running. That indicates factual use at that moment and was viewed as an intention to use CGs during the whole follow-up period. However, both the type of CG and the actual use of CGs after baseline were not reported in the observational study performed. Therefore, it was impossible to perform a per-protocol analysis and therefore a modified intention-to-treat analysis was chosen.

Given the non-randomized treatment assignment in this emulation of the target trial, statistical adjustments were applied to reduce bias in the effectiveness estimates and to achieve exchangeability of the treatment groups. These adjustments are described in the following paragraph.

### Data analysis

Baseline characteristics of the runners are reported using means and standard deviations (SD) or numbers and percentages (as applicable). Between-group comparisons for the baseline characteristics were not performed, based on current recommendations from the updated CONSORT guideline [26]. In order to provide insight into the comparability of the group CG-users and non-CG-users standardized mean differences (SMD) between these groups were reported using the CG-users group as the reference. An SMD < 0.20 was considered as a small difference, 0 < .50 was considered a moderate difference, < 0.80 a large difference [27].

The primary outcome, any lower extremity RRI during any 2-week period, was analyzed using a mixed logistic regression model [4,28]. A random intercept was included to correct for multiple measurements per participant over time. Time was included as a categorical variable: the first measurement in time was excluded for each cohort separately, as all included runners had reported not having a lower extremity RRI at baseline. As an additional step, we also included intervention-by-time, in order to study whether the effect of the intervention varied over time, and calculated the p-value with a likelihood ratio test.

In addition to CG use (yes/no), we included the following potential confounders in the analysis, based on recent literature; 1) Utrecht Marathon event running distance (half/full marathon) [3], 2) lower extremity RRI in the 12 months prior to study participation (yes/no) [15,29,30], 3) previous experience with half or full marathons (having run ≥ 1 half marathon yes/no) [31]. These potential confounders were specified prior to the analyses.

As the time when the baseline questionnaire was completed and thus the length of the follow-up varied per cohort, we used the Utrecht Marathon running event as a common time point for all cohorts to reconstruct a common timeline for all runners.

We present the results of the analysis as odds ratios with 95% confidence intervals and p-values. To understand any clustering effects, we calculated the intraclass correlation coefficient (ICC). As a sensitivity analysis, we analyzed the effects of CGs separately for each of the four cohorts. All effects with a 2-sided p-value < 0.05 were considered significant. Analyses were performed with SAS v9.2 (SAS Institute Inc., Cary, NC, USA).

## Results

Of the 919 runners invited to participate in the SUMMUM-2017, 509 responded to the invitation and provided informed consent (Fig 1). Of these runners, a total of 329 completed the baseline characteristics section of the baseline questionnaire, indicated that they subscribed for the Utrecht half or full marathon, and reported no RRI at baseline and were included in this study. The other 180 runners were excluded because they did not complete the baseline questionnaire (n = 40), registered for a running event other than the half or full Utrecht Marathon (n = 20), or reported a RRI at baseline (n = 120).

### Baseline characteristics and response rate

Of the 329 runners included in the emulation of the target trial, 267 (81.2%) registered for the half marathon and 62 (18.8%) for the full marathon (Table 2). CGs were used by 70 (21.3%) runners. Lower extremity RRIs in the 12-months before the start of this study were reported by 82 (24.9%) runners. The most frequently reported lower-extremity RRIs during this 12-months period affected the lower leg (n = 27; 32.5%) and knee (n = 25; 30.1%). The response rate in all cohorts ranged from 62% to 91% (Appendix 1). Based on the SMDs between the groups of CG-users and non-CG-users there were only small differences between these groups on gender, age, length, and training goal. A moderate between group difference was observed for weight and running experience. The group non-CG-users had a higher average weight and more often reported running experience than the group of CG-users.

**Table 2:** Baseline characteristics.

	Total cohort 4Mo-1Mo (n=329)		SMD between CG-users and non-CG-users^5^	Cohort 4Mo (n= 125)		Cohort 3Mo (n=41)		Cohort 2Mo (n=77)		Cohort 1Mo (n=86)	
Characteristics											
Gender, M/F n (%)	189/140	(57.4/42.6)	0.08	72/53	(57.6/41.5)	24/17	(58.5/41.5)	45/32	(58.4/41.6)	48/38	(55.8/44.2)
Age, years, mean (SD)	38.8	(11.5)	-0.14	38.1	(11.8)	40.2	(11.8)	38.4	(11.8)	39.7	(10.7)
Length, cm, mean (SD)	178.3	(8.9)	-0.16	178.2	(9.6)	180.1	(9.5)	178.6	(8.6)	177.6	(7.9)
Weight, kg, mean (SD)	72.0	(10.8)	-0.23	71.5	(11.2)	72.0	(11.3)	73.7	(11.8)	71.3	(8.7)
Training goal, 21km^1^/42km^2^ n (%)	267/62	(81.2/18.8)	0.07	100/25	(80.0/20.0)	32/9	(78.0/22.0)	68/9	(88.3/11.7)	67/19	(77.9/22.1)
Experienced runners^3^, n (%)	240	(72.9)	-0.23	89	(71.2)	32	(78.0)	52	(67.5)	67	(77.9)
Compression garment users, n (%)	70	(21.3)		26	(20.8)	12	(29.3)	12	(15.6)	20	(23.3)
Runner reporting a RRI during the 12-months before baseline, n (%)	82	(24.9)	0.15	40	(32.0)	9	(22.0)	15	(19.5)	18	(20.9)
Location of RRI during the 12-months before baseline^4^, n(%)											
Head or neck	3	(3.6)		1	(2.5)	1	(11.1)	1	(6.7)	0	
Upper extremity	2	(2.4)		1	(2.5)	0		1	(6.7)	0	
Upper back	1	(1.2)		1	(2.5)	0		0		0	
Lower back	7	(8.4)		2	(5.0)	1	(11.1)	2	(13.3)	2	(10.5)
Hip	6	(7.2)		3	(7.5)	1	(11.1)	1	(6.7)	1	(5.3)
Groin	2	(2.4)		1	(2.5)	0		1	(6.7)	0	
Buttock	6	(7.2)		2	(5.0)	0		3	(20.0)	1	(5.3)
Ventral side of the Upper leg	2	(2.4)		2	(5.0)	0		0		0	
Dorsal side of the upper leg	9	(10.8)		3	(7.5)	1	(11.1)	3	(20.0)	2	(10.5)
Knee	25	(30.1)		13	(32.5)	4	(44.4)	4	(26.7)	4	(16.0)
Lower leg	27	(32.5)		9	(22.5)	3	(33.3)	7	(46.7)	8	(41.1)
Achilles tendon	8	(9.6)		3	(7.5)	0		2	(13.3)	3	(15.8)
Ankle	10	(12.0)		8	(20.0)	0		1	(6.7)	1	(5.3)
Foot/toe	16	(19.3)		8	(20.0)	1	(11.1)	3	(20.0)	4	(21.1)

Abbreviations: %, percentage; cm, centimetres; F, female; kg, kilogram; km, kilometre, M, male; Mo, months; n, number; RRI, running-related injury; SD, standard deviation; SMD, standardized mean difference.

^1^Training goals is a half marathon (21 km);

^2^Training goal is a marathon (42 km).

^3^A runner was considered as experienced if he/she ran ≥ 1 half marathon.

^4^Runners could report multiple locations of RRIs during the 12-months before baseline.

^5^CG-users group was used as a reference for the standardized mean differences.

### Running related injuries

Table 3 shows the characteristics of the RRIs recorded during preparation for the Utrecht Marathon. The most frequently reported RRIs were located at the knee (n = 76; 36.7%), lower leg (n = 37; 17.9%), and dorsal side of the upper leg (n = 13; 6.3%). The most reported types of RRIs were overuse RRIs (n = 99; 47.8%) and ‘muscle or tendon RRIs’ (n = 58; 28.0%).

**Table 3:** Characteristics of the running-related injuries during the preparation period before the Utrecht Marathon.

	All cohorts	
RRI location, n (%)	172	
Hip	8	(3.9)
Groin	7	(3.4)
Buttock	3	(1.4)
Upper leg	9	(4.3)
Dorsal side of the upper leg	13	(6.3)
Knee	76	(36.7)
Lower leg	37	(17.9)
Achilles tendon	7	(3.4)
Ankle	12	(5.8)
Foot/toe	0	
Other	0	
Type of RRIs, n (%)	207	
Bursitis	0	
Cartilage injury	2	(1.0)
Contusion	6	(2.9)
Joint distortion	14	(6.8)
Fracture	0	
Joint Luxation	0	
Muscle or tendon RRI	58	(28.0)
Nerve injury	4	(1.9)
Overuse injury	99	(47.8)
Other	24	(11.6)

Abbreviations: %, percentage; n, number; RRI, running-related injury.

### Effects of compression garments

The results of the emulation of the target trial (Table 4) showed that the use of CGs did not significantly alter the odds of a lower extremity RRI at any time in half or full marathon runners during their preparation for the Utrecht Marathon (odds ratio 1.07 95% CI 0.48–2.40). The ICC for the mixed-model logistic regression was 0.56. The intervention-by-time interaction analysis was not significant and did not improve the model fit (*p* = 0.47). Therefore, we did not use this intervention-by-time interaction analysis for the outcome of the emulation.

**Table 4:** Effects of CGs on the occurrence of RRIs compared to no CGs based on the mixed logistic regression model with odds ratios for CG use and confounders.

Variables	Odds ratio^¶^ (95% CI)		*P*-value
CG use (CGs versus no-CGs^¥^)	1.07	(0.48 - 2.40)	0.86
Utrecht Marathon event running distance (half marathon Utrecht Marathon event running distance versus full marathon^¥^)	1.06	(0.45 - 2.52)	0.89
Lower extremity RRI reported in the 12 months prior to the participation in this study (yes versus no^¥^)	1.44	(0.68 - 3.04)	0.34
Previous experience with half or full marathons, having run ≥ 1 half marathon (yes versus no^¥^)	0.40	(0.19 - 0.84)	0.016
Time			
14 weeks before the Utrecht Marathon	0.21	(0.09 - 0.50)	0.0005
12 weeks before the Utrecht Marathon	0.18	(0.08 - 0.44)	0.0001
10 weeks before the Utrecht Marathon	0.32	(0.15 - 0.70)	0.0041
8 weeks before the Utrecht Marathon	0.14	(0.06 - 0.30)	<0.0001
6 weeks before the Utrecht Marathon	0.51	(0.27 - 0.95)	0.0331
4 weeks before the Utrecht Marathon	0.41	(0.23 - 0.73)	0.0025
2 weeks before the Utrecht Marathon	0.65	(0.37 - 1.12)	0.12
week of the Utrecht Marathon *	0	.	.

Abbreviations: 95% CI, 95% confidence interval; CG, compression garments; RRI, running-related injury; UM, Utrecht Marathon.

¥ reference category denotes the category that was scored as “0” for a specific variable in the regression equation.

¶ Higher odds ratio indicates a greater risk of lower extremity RRIs

*reference category for the time variable, denotes the category that was scored as “0” for a specific variable in the regression equation.

### Sensitivity analyses

We analyzed each cohort separately in a sensitivity analysis. Appendix 2 shows the odds ratios per cohort. The odds ratio for cohort 3Mo showed a significant effect in favor of CGs (OR 0.07, 95% CI 0.006–0.80, p = 0.0325). For the cohorts 4Mo, 2Mo and 1Mo no significant findings were found.

## Discussion

This study assessed the effectiveness of CGs on the occurrence of lower extremity RRIs in half and full marathon runners who were injury-free at baseline during their preparation for the 2017 Utrecht Marathon. Because primary and secondary prevention of RRIs are the most important reasons for runners to use CGs [8], we attempted to emulate an imaginary RCT using target trial emulation methodology. Based on our analyses no significant positive or negative effects of CG use on the odds of lower extremity RRIs were identified in the mixed logistic regression model analysis.

If data on the compliance of CG use, pressure gradient, types of CG used and external training load were available within the SUMMUM-2017 study we might have been able to make a more accurate effect estimate for CG use. However, as the most mentioned primary reason for endurance athletes to use CGs is sports injury prevention and that to our best knowledge there have been no RCTs on the effects of CGs on RRI recurrence or prevention in athletes who participate in sports that require any level of running [8]. We believe that our results are of importance in order to highlight this important gap of knowledge and show the potential of target trial emulations in sports medicine by attempting to apply it to the use of CGs.

The lower extremity sports injuries for which endurance athletes primarily use CGs are mainly lower leg injuries (64.8%), specifically located at the shin or calf [8]. An example of such a sports injury is medial tibial stress syndrome (MTSS) [32]. Several RCTs have studied the effectiveness CGs as adjunctive therapy for MTSS in athletes [33] and military personnel [34–36], but with conflicting results. Our study corroborates the findings of Moen et al. (2012), because we found no significant effects of CGs on the risk of lower extremity sports injuries in athletes preparing for a half or full marathon.

Although the precise physiological effects of CGs remain the subject of further investigation, a narrative review of Brown et al. (2023) proposed an addition to previously hypothesized mechanisms for recovery after sports by using CGs [37]. They reported that positive effects of CGs are found when the compression provided by the CGs is sufficient to decrease muscle edema (measured as limb circumference) as a result of exercise-induced muscle damage. In order to exert an effect on muscle edema and local hemodynamics, the CG pressure at the calf should hypothetically be between 19 and 24 mmHg [37,38]. These findings are made possible by the development of more accurate measurement of in vivo CG pressure by portable pressure monitors [37,39]. Regarding the effects of CGs on the occurrence of lower extremity RRIs, Brown et al. (2023) suggests that only CGs which provide sufficient pressure to decrease muscle edema can influence the occurrence of RRIs. Further, if muscle edema is decreased, muscle adaptation and functional recovery might hypothetically occur more quickly compared to non-CGs or placebo CGs. A speedy recovery after training or an event is probably important for endurance athletes, such as runners, because it might decrease the chance of overload RRIs, which are the most frequently occurring type of RRIs in runners [4,40]. Previously, Bovenschen et al. (2013) reported that CGs which provided 25–35 mmHg pressure to no CGs were able to significantly reduce leg volume after a 10 km running test and treadmill maximum exercise test compared [6]. These aforementioned hypotheses on the dose-response mechanism for CGs and the effects of CGs on the occurrence of lower extremity RRIs should be of interest to investigate further in future studies that (periodically) measure and check the in vivo pressure provided by CGs. Other studies have also explored the effects of compression garments on biomechanical variables. However, a recent systematic review showed that there is no evidence that the use of CGs influence the occurrence of lower extremity sports injuries including RRIs [15].

Target trial emulation methodology is becoming increasingly popular to assess the relative effectiveness of interventions and can be placed in a broader development wherein trials can be performed both in the real-world and virtual [41,42]. This method provides researchers with the possibility to evaluate interventions precisely as they would be applied in the real world and thus gain an understanding of the intervention which would not be obtained through an RCT [20,43]. The Cochrane review of Anglemyer et al. (2014) might have contributed to the popularity of target trial emulations as they showed that the estimates attained from observational studies on the comparative effectiveness of interventions can be valid [43]. Anglemeyer et al. (2014) reasoned that even though observational studies may be more biased than RCTs, the analyses do not result in larger or smaller intervention effects. As such, target trial emulation methodology could be used more often in order to make more use of data from observational studies and improve the quality of analyses by explicating the causal framework used [20,22].

To the best of our knowledge, there are only a few prospective (observational) studies of CG use. For example, Lucas-Cuevas et al. (2015) studied the effects of CGs on stride kinematics, impact acceleration, and perceived comfort over 3 weeks [16]. In-vivo CG pressure and compliance with the intervention were not measured. Unfortunately, as in our study, none of the other studies measured CG pressure or registered compliance with CG use during the follow-up period, making it impossible to study differences between full and partial CG use. Consequently, also in our study, a firm conclusion whether the self-selected CGs are effective to prevent RRIs is hard to make, and the null results cannot be interpreted as a fact that compression in itself would have no effect on the prevention of RRIs. Potentially, runners who use their CGs more often and at the right moment have a greater benefit from using their CGs.

### Limitations

This study had several limitations. In the SUMMUM-2017 study, we asked all runners who registered for the half and full Utrecht Marathon to participate but only 329 met inclusion criteria for the target trial emulation. So, the sample size of our study is limited, which is reflected in the wide confidence intervals, and we did not perform any sample size calculation as would have been performed for a RCT. Alternatively, we calculated a minimal detectable effect of approximately 0.36. This is based on the incidence of injury in non-CG users, the total number participants in the SUMMUM-2017 study and the proportion of these participants using a CG and the ICC as reported in our study.

Initial results suggested that some participants started using CGs due to an existing RRI, i.e., a RRI was present at baseline. As an existing RRI may result in a recurrence or higher probability of new RRIs, these participants were excluded. Further, for the sensitivity analyses the sample size in each cohort is relatively small, as is reflected by the wide 95% confidence intervals for the odds ratios. The significant effect at 3Mo was based on only 41 participants and may represent an accidental finding. Based on the limited sample size of our study we also chose not to include covariates such as body mass index and age for which the data was available. There was no data, within the SUMMUM-2017 study, on covariates of interest such as internal (such as rated perceived exertion [44]) and external training load (per example weekly training milage), CG pressure gradient, the use of concurrent prevention strategies (per example strength training). In order to optimize statistical power for the main analysis, we applied a random effect model that allowed us to combine all cohorts and all available data. As the study did not contain data on all potential confounders, there is a risk of residual confounding.

Within the SUMMUM-2017 study CG users reported which CGs they used via an online questionnaire. The CG pressure gradient was not reported in the online questionnaire and furthermore we were unable to measure the type of CG used and the CG pressure gradient in-vivo. Thus, no data was availability on the reliability of the exposure. Ideally, the in-vivo CG pressure would be measured multiple times as mentioned above CG pressure might relate to the physiological effects [37,38]. Further, we were unable to estimate the test-retest reliability of the Dutch version of the OSTRC questionnaire used because there were insufficient runners whose injury status was unchanged in two consecutive measurements. Nonetheless, test-retest reliability of the OSTRC questionnaire on health problems in other languages is reported in three studies. For the Thai version (first version of the OSTRC on health problems) a test-retest reliability intraclass correlation coefficient (ICC) of 0.970, 95% CI 0.950 to 0.982 in 57 thai athletes was reported [45]. For the Greek version of the second version of the OSTRC a test-retest reliability ICC of 0.93, 95% CI 0.89–0.97, in 56 female volleyball athletes was found [46]. And for the Persian version (OSTRC-second version) reported a test-retest reliability ICC range: 0.82–1.00 in 207 elite Persian athletes from various sports [47].

We did not include internal or external training load in our target trial emulation. Recent statements on methodology in sports medicine have highlighted the importance of measuring training load and recommend measuring absolute or relative changes training load over time [48,49]. More experienced runners are probably better able to regulate their training load due to their experience and thus their RRI risk will decrease [3,50,51]. In order to measure internal and external training load in runners and other athletic populations respectively rated-perceived exertion and GPS-data could be used [49,52,53]. However, as training load was not registered in the SUMMUM-2017 study, in our target trial emulation, we decided to use running experience (having run ≥ 1 half marathon yes/no) as a proxy and added it as a confounder.

A third limitation is that our study used a time axis which was reconstructed in a negative direction. Within observational studies and RCTs with multiple follow-up measurements, starting with the baseline measurements, are often performed. During the analyses of the data these measurements are used in sequence to reconstruct a timeline. Within the present observational study we allowed for runners to enter our study at multiple time points, specifically 16 weeks, 12 weeks, 8 weeks, and 4weeks before the Utrecht Marathon. Consequently, the common measurement in time for all runners was the last one just before the Utrecht Marathon. So, for the analysis we reconstructed the time axis in a negative direction, backwards from the Utrecht Marathon to the follow-up period in order to build a common timeline for all runners. With a somewhat different design with a common and extensive baseline measurement for all runners and higher numbers, especially for CG use, propensity score analyses would have been a better option. Such analyses generally would have led to improved exchangeability between the CG users and non-CG users by including more confounders, and results in marginal treatment effects [54]. Thus, having a common baseline measurement for all participants and extensive measurements of potential confounders is recommended.

As previously stated, the aim of this study was to use and approximate target trial emulation methodology as much as possible, given the (methodological) limitations of our study. Nonetheless, we believe that our results are important to both CG-users and sports medicine researchers because they show the potential and complexity of performing target trial emulations based on our real-world experience. In order to clarify the use of target trial emulation methodology by using data from observational studies, we provide an overview of the necessary steps and considerations to perform a target trial emulation (Table 1). The nine included steps help guide the preparation of the observational study and facilitate the emulation. It is important that researchers who consider target trial emulations collect data regarding the intervention used, the event of interest, and potential confounders and effect modifiers.

## Conclusion

We studied the use of a self-selected CG in half and full marathon runners, who had no RRIs at baseline, on the occurrence of lower extremity RRIs during their preparation for the 2017 Utrecht Marathon running event. We identified no significant effects of CGs on the occurrence of lower extremity RRIs in these runners. However, because no compression grade of compliance was registered in this study, it does not automatically mean that compression in itself would have no effect on prevention of RRIs. Furthermore, this study shows how target trial emulation methodology can be used to assess the relative effectiveness of interventions and can increase the utility of data attained from observational studies. Future studies, both observational studies and RCTs, on the incidence of (recurring) RRIs in both uninjured and injured half and full marathon runners are needed. By also investigating the effects of CGs while (periodically) measuring the in vivo pressure provided by the CGs, and compliance, these studies will further contribute to answer the question whether CGs can help to prevent and reduce RRIs.

What is already known on this topic:

- Running is a popular sport for millions across the world. Amongst these runners compression garments (CGs) are used often.
- CGs are primarily used to reduce the risk of lower extremity running-related injuries (RRIs).
- Studies on the effects of CGs on lower extremity RRIs are scarce.

What this study adds:

- We studied the effects of CGs on the risk of lower extremity RRIs by approximating target trial emulation methodology.
- No evidence for a beneficial or detrimental effect of CGs on the risk of lower extremity RRIs was found in half and full marathon runners (odds ratio 1.07 95% CI 0.48–2.40).

How this study might affect research, practice or policy

- These findings provide insight for CG users and sports medicine clinicians regarding the effectiveness of CGs and their use for RRI prevention in daily practice.
- Our findings warrant future studies on the effects of CGs with periodic measurement of in vivo pressure and compliance on the occurrence of RRIs.

## Supporting information

S1 Appendix 1. Table Response rate of the SUMMUM-2017 biweekly online questionnaire for all runners per cohort (N = 329). Abbreviations; %; percentage, Mo; months, n; number, wk; weeks, UM; Utrecht Marathon.
(DOCX)
