# Supplementary material for: The effectiveness of compression garments on RRI prevention in half- and full-marathon runners: Emulation of a RCT using data from an observational study
Source: PLoS One. 2026 Nov 12;21(11):e0362483. doi: 10.1371/journal.pone.0362483 (PMC13759087; doi:10.1371/journal.pone.0362483)
Supplement: Supplementary file 1 [file pone.0362483.s001.docx]

**Appendix**

**Appendix 1** Response rate of the SUMMUM-2017 biweekly online questionnaire for all runners per cohort (N=329)

| Cohort  Wk before UM | 4Mo, n(%) | | 3Mo, n(%) | | 2Mo, n(%) | | 1Mo, n(%) | |  |
| --- | --- | --- | --- | --- | --- | --- | --- | --- | --- |
| 16 | 125 | (100) |  |  |  |  |  |  | |
| 14 | 108 | (86) |  |  |  |  |  |  | |
| 12 | 89 | (71) | 41 | (100) |  |  |  |  | |
| 10 | 84 | (67) | 37 | (90) |  |  |  |  | |
| 8 | 87 | (70) | 34 | (83) | 77 | (100) |  |  | |
| 6 | 79 | (63) | 34 | (83) | 68 | (88) |  |  | |
| 4 | 86 | (69) | 33 | (80) | 67 | (87) | 86 | (100) | |
| 2 | 77 | (62) | 33 | (80) | 64 | (83) | 73 | (85) | |
| 0 | 94 | (75) | 35 | (85) | 70 | (91) | 71 | (83) | |

Abbreviations; %; percentage, Mo; months, n; number, wk; weeks, UM; Utrecht Marathon

**Appendix 2** Sensitivity analyses to study the odds of a lower extremity RRI during the preparatory period before the half or full Utrecht Marathon per cohort

| **Cohort** | **Odds ratio (95% CI)** | |
| --- | --- | --- |
| Cohort 4Mo^¶^ | 2.94 | (0.67; 12.95) |
| Cohort 3Mo^¶^ | 0.07 | (0.006; 0.80) |
| Cohort 2Mo^¶^ | 0.96 | (0.19; 4.93) |
| Cohort 1Mo^¶^ | 2.2 | (0.007; 687.05) |

Abbreviations; CI, confidence interval; Mo, months

^¶^ indicates the treatment effects of CGs where higher odds ratio indicates a greater risk of lower extremity RRIs
