# Supplementary material for: The effectiveness of compression garments on RRI prevention in half- and full-marathon runners: Emulation of a RCT using data from an observational study
Source: PLoS One. 2026 Nov 12;21(11):e0362483. doi: 10.1371/journal.pone.0362483 (PMC13759087; doi:10.1371/journal.pone.0362483)
Supplement: Supplementary file 2 [file pone.0362483.s003.docx]

When submitting your revision, we need you to address these additional requirements.

1. Please ensure that your manuscript meets PLOS ONE's style requirements, including those for file naming. The PLOS ONE style templates can be found at

<https://journals.plos.org/plosone/s/file?id=wjVg/PLOSOne_formatting_sample_main_body.pdf> and

[https://journals.plos.org/plosone/s/file?id=ba62/PLOSOne_formatting_sample_title_authors_affiliations.pdf](https://track.editorialmanager.com/CL0/https:%2F%2Fjournals.plos.org%2Fplosone%2Fs%2Ffile%3Fid=ba62%2FPLOSOne_formatting_sample_title_authors_affiliations.pdf/1/010f019cdde57f0c-2a97a394-99de-4771-8acb-ce07fa416be1-000000/WuO0hbF8GDLLX6v8-bEl9A3cIH5O3jyvscSnfWIDAf4=254)

2. Thank you for stating the following in the Competing Interests section:
NO authors have competing interests

Please confirm that this does not alter your adherence to all PLOS ONE policies on sharing data and materials, by including the following statement: "This does not alter our adherence to PLOS ONE policies on sharing data and materials.” (as detailed online in our guide for authors <http://journals.plos.org/plosone/s/competing-interests>). If there are restrictions on sharing of data and/or materials, please state these. Please note that we cannot proceed with consideration of your article until this information has been declared.

Please include your updated Competing Interests statement in your cover letter; we will change the online submission form on your behalf.

3. In this instance it seems there may be acceptable restrictions in place that prevent the public sharing of your minimal data. However, in line with our goal of ensuring long-term data availability to all interested researchers, PLOS’ Data Policy states that authors cannot be the sole named individuals responsible for ensuring data access (<http://journals.plos.org/plosone/s/data-availability#loc-acceptable-data-sharing-methods>).

Data requests to a non-author institutional point of contact, such as a data access or ethics committee, helps guarantee long term stability and availability of data. Providing interested researchers with a durable point of contact ensures data will be accessible even if an author changes email addresses, institutions, or becomes unavailable to answer requests.

Before we proceed with your manuscript, please also provide non-author contact information (phone/email/hyperlink) for a data access committee, ethics committee, or other institutional body to which data requests may be sent. If no institutional body is available to respond to requests for your minimal data, please consider if there any institutional representatives who did not collaborate in the study, and are not listed as authors on the manuscript, who would be able to hold the data and respond to external requests for data access? If so, please provide their contact information (i.e., email address). Please also provide details on how you will ensure persistent or long-term data storage and availability.

4. Please include captions for your Supporting Information files at the end of your manuscript, and update any in-text citations to match accordingly. Please see our Supporting Information guidelines for more information: <http://journals.plos.org/plosone/s/supporting-information>.

5. If the reviewer comments include a recommendation to cite specific previously published works, please review and evaluate these publications to determine whether they are relevant and should be cited. There is no requirement to cite these works unless the editor has indicated otherwise.

[Note: HTML markup is below. Please do not edit.]

**Detailed comment response**
Reviewers' comments:

**Comment 1**

Reviewer's Responses to Questions

1. Is the manuscript technically sound, and do the data support the conclusions?

The manuscript must describe a technically sound piece of scientific research with data that supports the conclusions. Experiments must have been conducted rigorously, with appropriate controls, replication, and sample sizes. The conclusions must be drawn appropriately based on the data presented.

Reviewer #1: Partly

Reviewer #2: Partly

2. Has the statistical analysis been performed appropriately and rigorously?

Reviewer #1: Yes

Reviewer #2: No

3. Have the authors made all data underlying the findings in their manuscript fully available?

The [PLOS Data policy](http://track.editorialmanager.com/CL0/http:%2F%2Fwww.plosone.org%2Fstatic%2Fpolicies.action%23sharing/1/010f019cdde57f0c-2a97a394-99de-4771-8acb-ce07fa416be1-000000/HWrp0RGFC6etnIwgf69LhPw0grNGL52TSPXoEVsRja8=254) requires authors to make all data underlying the findings described in their manuscript fully available without restriction, with rare exception (please refer to the Data Availability Statement in the manuscript PDF file). The data should be provided as part of the manuscript or its supporting information, or deposited to a public repository. For example, in addition to summary statistics, the data points behind means, medians and variance measures should be available. If there are restrictions on publicly sharing data—e.g. participant privacy or use of data from a third party—those must be specified.

Reviewer #1: Yes

Reviewer #2: No

4. Is the manuscript presented in an intelligible fashion and written in standard English?

PLOS ONE does not copyedit accepted manuscripts, so the language in submitted articles must be clear, correct, and unambiguous. Any typographical or grammatical errors should be corrected at revision, so please note any specific errors here.

Reviewer #1: Yes

Reviewer #2: Yes

5. Review Comments to the Author

Please use the space provided to explain your answers to the questions above. You may also include additional comments for the author, including concerns about dual publication, research ethics, or publication ethics. (Please upload your review as an attachment if it exceeds 20,000 characters)

Reviewer #1: The effectiveness of compression garments on RRI prevention in half- and full-marathon runners: emulation of a RCT using data from an observation study"
This manuscript investigates an important and practical question regarding the preventive effects of compression garments (CGs) on running-related injuries (RRIs) in marathon runners. The authors employ an innovative target trial emulation approach using observational data from the SUMMUM-2017 study, addressing a gap in the literature where randomized controlled trials are lacking.

**Response 1**

We thank the reviewers for the time they invested in providing feedback on our manuscript. In the comment-response below we will describe our response and the changes we made to our manuscript.

**Comment 2**
Strengths and Weaknesses
The study demonstrates several notable strengths. The application of target trial emulation methodology represents a sophisticated approach to causal inference from observational data, providing a framework that could be valuable for future sports medicine research. The authors transparently report their methodology through a comprehensive table outlining the nine components of their emulation approach, which enhances reproducibility. The research question addresses a real-world concern for runners and clinicians, as CGs are widely used despite limited evidence for injury prevention. The statistical approach using mixed logistic regression with random intercepts appropriately accounts for the repeated measures design.
However, several limitations compromise the study's impact. The sample size of 329 participants appears insufficient, particularly given the wide confidence intervals (OR 1.07, 95% CI 0.48-2.40) that suggest the study was underpowered to detect clinically meaningful effects. The lack of measurement of in vivo CG pressure represents a critical omission, as recent literature suggests pressure gradients between 19-24 mmHg are necessary for physiological effects. The absence of training load data is problematic, as this is a key confounder in running injury research. The retrospective timeline reconstruction, while creative, introduces potential bias that differs from prospective data collection. Furthermore, the reliance on self-reported injury data without clinical verification may affect outcome accuracy.

**Response 2**

We thank reviewer 1 for their appreciation of our manuscript. We agree that our manuscript has several limitations. The limitations mentioned here are also acknowledged by the authors in the limitations section of the manuscript

1. The sample size is limited indeed (n=329). We have noted our limited sample size on line 343-345.
   “So, the sample size of our study is limited, which is reflected in the wide confidence intervals, and we did not perform any sample size calculation as would have been performed for a RCT.”
2. We agree with the reviewer and have discussed the reference to which the reviewer refers on the relationship between the pressure gradient and the physiological effects of CGs. We have now added the following text tot the limitations section.
   “Within the SUMMUM-2017 study CG users reported which CGs they used via an online questionnaire. We could not measure the in-vivo CG pressure. Ideally, the in-vivo CG pressure would be measured multiple times as mentioned above CG pressure might relate to the physiological effects.” (line 357-361)
3. Training load data
   On this point we agree with the reviewer as well. Therefore, we added the following text to the limitations section on line 362-371.
   “We did not include training load in our target trial emulation. Recent statements on methodology in sports medicine recommend measuring absolute or relative changes training load over time.(39) More experienced runners are probably better able to regulate their training load due to their experience and thus their RRI risk will decrease.(3,40,41) In order to measure internal and external training load in runners respectively rated-perceived exertion and GPS-data could be used. (42, 45) However, as training load was not registered in the SUMMUM-2017 study, in our target trial emulation, we decided to use running experience (having run ≥1 half marathon yes/no) as a proxy and added it as a confounder.”
4. Negative timeline
   We thank the reviewer for recognizing our creative solution. However, we would like to clarify that the data collection for the SUMMUM-2017 study was performed prospectively. The analysis for our target trial emulation were performed with a negative reconstruction in order to create a common point in time in which all the cohorts are present.
   In order to clarify this nuance we added the yellow marked text to
   Table 1, item 2 “study design”, column “part 1 SUMMUM-2017”, we added “prospective” to the study design.
   line 378-380 “So, for the analysis we reconstructed the time axis in a negative direction, backwards from the Utrecht Marathon to the follow-up period in order to build a common timeline for all runners.”
5. Self reported data
   Within sports medicine research and specifically the research on running-related injuries (RRIs) self-reported (online) questionnaires are a common method of collecting data and following athletes for a longer period of time. Examples of observational studies are [Kluitenberg et al. 2019](https://pubmed.ncbi.nlm.nih.gov/29934211/), [Fokkema et al. 2019](https://pubmed.ncbi.nlm.nih.gov/30268637/) .[Schuster Brandt Fernsten et al. 2025](https://pubmed.ncbi.nlm.nih.gov/40623829/).

Further, during the period we designed the SUMMUM studies the consensus statement from Timpka et al. (BJSM 2014) was current. Although the statement acknowledges the use of diagnoses and reporting by qualified medical personnel it also acknowledges the use of self-reported data as a suitable alternative.

**Comment 3**

Statistical Considerations
The statistical analysis raises concerns about adequate power. With only 70 CG users (21.3%) among 329 participants, detecting moderate effect sizes would be challenging. A post-hoc power calculation would strengthen the manuscript. The authors should report the intraclass correlation coefficient from their mixed model to understand the clustering effect. Additionally, the number of events (injuries) per predictor variable should be reported to assess model stability - generally, 10-15 events per variable are recommended for logistic regression.

**Response 3**

We have calculated the ICC for the mixed model analysis and added it to the manuscript on line

Line 217 “To understand any clustering effects, we calculated the intraclass correlation coefficient (ICC).”

Line 265--266 “The ICC for the mixed-model logistic regression was 0.56.”

We agree with the reviewer that our sample size is relatively low. However, we have not added a post-hoc power analysis as this is not advised in the literature, see for example [Heinsberg et al. 2023](https://pmc.ncbi.nlm.nih.gov/articles/PMC9452450/#:~:text=observed%20statistical%20results.-,We%20encourage%20authors%20and%20peer%2Dreviewers%20to%20avoid%20using%20or,they%20are%20simply%20not%20informative.), Zhang 2019, [Hoenig & Heisey (2001)](https://sciences.ucf.edu/biology/pascencio/wp-content/uploads/sites/24/2016/11/HoenigHeisey2001.pdf), [Levine & Ensom (2001)](https://europepmc.org/article/MED/11310512).

Levine & Ensom 2001 reported that it is preferred to use the 95% confidence interval (CI) in order to inform our readers regarding appropriateness of our sample size for the analysis performed. Within our limitations section we admit that the limitations of our sample size is reflected in the wide confidence intervals (line 343-345).

“So, the sample size of our study is limited, which is reflected in the wide confidence intervals, and we did not perform any sample size calculation as would have been performed for a RCT.”

**Comment 4**
Quality Assessment
The tables are generally well-constructed, though Table 2 could benefit from reporting standardized differences between CG users and non-users to assess baseline balance. Figure 1 (flowchart) effectively illustrates participant flow but could include reasons for loss to follow-up. The English is generally clear, though some sentences are overly complex and could be simplified for international readers.

**Response 4**

We have added the standardized differences to table 2 on line 243. Furthermore, we have added the following text to the data-analysis paragraph on line 198-201 “In order to provide insight into the comparability of the group CG-users and non-CG-users standardized mean differences (SMD) between these groups were reported using the CG-users group as the reference. An SMD <.2 was considered as a small difference, <.5 was considered a moderate difference, <.8 a large difference. (26)”

Unfortunately, the observational study did not collect data on reasons for loss to follow-up. Therefore, we cannot report this.

**Comment 5**
Recommended References
To strengthen this manuscript, I recommend incorporating the following references at strategic points:
1.In the Introduction (after discussing CG mechanisms): The discussion of physiological mechanisms would benefit from citing Padulo et al.'s work on biomechanical aspects: "The acute effect of whole-body vibration on repeated shuttle-running in young soccer players" (Padulo J, et al. Int J Sports Med. 2014;35(1):49-54), which provides insights into how external interventions may affect running mechanics and injury risk.

**Response 5**

We agree that the biomechanics of the external interventions such as CGs are important. However, specifically for CGs much is still unknown. Therefore, we have added, to this manuscript, a reference to our previously published systematic review. ([Franke et al. 2024](https://pubmed.ncbi.nlm.nih.gov/38665681/)) The conclusion from our systematic review was “We identified no evidence for a beneficial or detrimental effect of lower leg CGs on the occurrence of lower extremity sports injuries, subjective ratings of fatigue, or biomechanical variables in athletes at any level of running performance.”

We have added the reference to this manuscript on line 76-78 “The precise physiological and biomechanical mechanisms by which CGs stimulate post-exercise recover are not fully understood.”

**Comment 6**
2.In the Methods section (when discussing injury definitions): The injury definition and classification system could be strengthened by referencing the consensus work: "I.S.Mu.L.T - Rotator cuff tears guidelines" (Oliva F, et al. Muscles Ligaments Tendons J. 2015;5(4):227-263), which, while focused on upper extremity, provides a framework for standardizing injury definitions in sports medicine research.

**Response 6**

We agree with the reviewer that the article by Oliva et al. (2016) provides a helpful framework in the upper extremity field. However, we used another framework as we used the consensus statement from Timpka et al (2014), which was current at the time we performed our data collection. Moreover, the definitions and methodology we used were in line with other prospective cohort studies in our field and our prior SUMMUM-2016 study (Franke et al 2019, van Poppel et al 2014, Kluitenberg et al. 2015)

**Comment 7**

3.In the Discussion (when addressing training load): The limitation regarding unmeasured training load should reference: "Statement on methods in sport injury research from the 1st METHODS MATTER Meeting, Copenhagen, 2019" (Nielsen RO, et al. Br J Sports Med. 2020;54(15):941), which explicitly addresses the importance of load monitoring in injury research.

**Response 7**

We have altered the sentence on line 362-365 to “Recent statements on methodology in sports medicine have highlighted the importance of measuring training load and recommend measuring absolute or relative changes training load over time. (41, 42) ”

**Comment 8**
4.In the Discussion (regarding CG pressure): The discussion of CG pressure requirements would be enhanced by citing: "Graduated compression stockings for runners: Friend, Foe, or Fake?" (Bovenschen JH, et al. J Athl Train. 2013;48(2):226-232), which provides critical analysis of compression garment efficacy.

**Response 8**

On line 312-315 we added a sentence that cites the proposed article.

**“**Previously, Bovenschen et al. (2013) reported that CGs which provided 25-35mmHg pressure to no CGs were able to significantly reduce leg volume after a 10km running test and treadmill maximum exercise test compared.”

**Comment 9**

5.In the Conclusion (regarding future research directions): The recommendations for future studies should reference: "The construct validity of session RPE during an intensive camp in young male Karate athletes" (Padulo J, et al. Muscles Ligaments Tendons J. 2014;4(2):121-126), which demonstrates validated methods for monitoring training intensity that could be applied to running studies.

**Response 9**

To the authors best knowledge it is not common practice to reference/cite manuscripts in the conclusion section. To verify our viewpoint we have checked 10 recent manuscripts from the sports science section from PlosOne. None of them cited other manuscripts in their conclusion section.

In order to address the point of future studies measuring internal training load (i.e. RPE) we have added the following sentence on line 366-368 “In order to measure internal and external training load in runners and other athletic populations respectively rated-perceived exertion and GPS-data could be used (Bourdon 2017 Padulo 2014)”.

**Comment 10**
Recommendations for Improvement
The authors should consider conducting a formal sample size calculation and reporting the observed power. Adding a supplementary analysis examining dose-response relationships (frequency of CG use if available) would strengthen the findings. The discussion would benefit from a more critical appraisal of why their findings differ from the biomechanical studies suggesting CGs reduce impact forces. Finally, the clinical implications should be stated more clearly - what should runners and clinicians conclude from these null findings?

**Response 10**

We refer the reviewer to response 3 regarding our response on post-hoc sample size calculations.

Unfortunately, we did not measure the frequency with which the runners used their CGs or the pressure gradient provided by the CGs within the SUMMUM-2017 study. Thus, we cannot perform the analysis suggested. In order to make this more clear within the manuscript we have added the yellow marked text to Table 1 row “4. Assignment procedures”, column “Part 1. SUMMUM-2017 study”: “At baseline all runners reported whether or not they use CGs. Runners chose themselves if and when they used their CGs.”

Furthermore, we have added the following sentences to the discussion section:

1. “These aforementioned hypotheses on the dose-response mechanism for CGs and the effects of CGs on the occurrence of lower extremity RRIs should be of interest to investigate further in future studies that (periodically) measure and check the in vivo pressure provided by CGs.”. (line 315-318)
   This sentence also highlights the importance of further research into the dose response relationship between the use of CGs, the pressure gradient and the occurrence of lower extremity RRIs.
2. “Within the SUMMUM-2017 study CG users reported which CGs they used via an online questionnaire. The CG pressure gradient was not reported in the online questionnaire and furthermore we were unable to measure the type of CG used and the CG pressure gradient in-vivo. Ideally, the in-vivo CG pressure would be measured multiple times as mentioned above CG pressure might relate to the physiological effects.” (line 357-361)

Regarding the biomechanical studies: We believe the reviewer is referring to manuscripts such as Lucas-Cuevas et al. 2015 and Broatch 2020. We have not discussed these references as we have discussed them in the systematic review that is already published and described the effects of CG use on the occurrence of sports injuries ([Franke et al. 2024](https://pubmed.ncbi.nlm.nih.gov/38665681/)) The conclusion from our systematic review was “We identified no evidence for a beneficial or detrimental effect of lower leg CGs on the occurrence of lower extremity sports injuries, subjective ratings of fatigue, or biomechanical variables in athletes at any level of running performance.”

To make this more clear we have added a sentence and reference on line 318 – 320 “Other studies have also explored the effects of compression garments on biomechanical variables. However, a recent systematic review showed that there is no evidence that the use of CGs influence the occurrence of lower extremity sports injuries including RRIs. (Franke 2024)”

**Comment 7**

Complete List of Suggested Citations
1.Padulo J, Di Giminiani R, Ibba G, Zarrouk N, Moalla W, Attene G, M. Migliaccio G, Pizzolato F, Bishop D, Chamari K. The acute effect of whole body vibration on repeated shuttle-running in young soccer players. International Journal of Sports Medicine. 2014;35(1):49-54.
2.Oliva F, Piccirilli E, Bossa M, et al. I.S.Mu.L.T - Rotator cuff tears guidelines. Muscles, Ligaments and Tendons Journal. 2015;5(4):227-263.
3.Nielsen RO, Shrier I, Casals M, et al. Statement on methods in sport injury research from the 1st METHODS MATTER Meeting, Copenhagen, 2019. British Journal of Sports Medicine. 2020;54(15):941.
4.Bovenschen JH, Te Booij M, Van Der Vleuten CJM. Graduated compression stockings for runners: Friend, Foe, or Fake? Journal of Athletic Training. 2013;48(2):226-232.
5.Padulo J, Chaabène H, Tabben M, et al. The construct validity of session RPE during an intensive camp in young male Karate athletes. Muscles, Ligaments and Tendons Journal. 2014;4(2):121-126.

**Response 7**

We refer the reviewer to our previous responses regarding suggested citations 1-5

**Comment 8**
Reviewer #2: The manuscript by Franke et al. explores the effectiveness of compression garments (CGs) on running-related injuries (RRIs) by emulating a hypothetical target trial using observational data from the 2017 Utrecht Half and Full Marathon. In general, I find the methodological approach to be as rigorous as possible given the constraints of the dataset, and I commend the authors for the transparency and honesty with which they discuss their biases and study limitations.

However, my primary concern lies in the substantial lack of control over the independent variable (CG use). While the authors attempt to mitigate uncertainty through a modified intention-to-treat approach, this strategy appears insufficient for a robust evaluation of CG effects. There is no available data regarding the compression dosage (mmHg), the specific type of garment used, the actual training mileage performed with or without CGs, or overall participant compliance. While I highly value the authors' efforts and the clarity of their reporting, I believe this absence of granular control over the intervention limits the capacity of the study to truly emulate a randomized controlled trial.

**Response 8**

We thank the reviewer for her/his efforts in and feedback on our manuscript.

We agree with the reviewer that the data collection of the SUMMUM-2017 study and our dataset has multiple limitations. We also agree that with the SUMMUM-2017 study there was no control over the independent variable (CG use). However, as mentioned in the methods section (paragraph Design of the SUMMUM-2017 study starting on line 101) and Table 1 the SUMMUM-2017 was an observational study and therefore we did not control any of the variables measured. Target trial emulation methodology is suitable for such analysis of data from observational studies and as it often is a secondary analysis of data previously collected the independent variable is not often controlled. ([Matthews et al. BMJ 2022](https://www.bmj.com/content/378/bmj-2022-071108)). However, we agree that more insight into the use of CGs by the runners, pressure gradients, types of CGs used and external training load is desirable. We therefore nuanced our findings in the discussion and conclusion section (see changes below). Furthermore, we made it more clear in Table 1 that we did not control the independent variable (see response 2 for the changes made to Table 1).

1. “Because primary and secondary prevention of RRIs are the most important reasons for runners to use CGs(7), we attempted to emulate an imaginary RCT using target trial emulation methodology.” (line 282)
2. “If data on the frequency of CG use, pressure gradient, types of CG used and external training load were available within the SUMMUM-2017 study we might have been able to make a more accurate effect estimate for CG use. However, as the most mentioned primary reason for endurance athletes to use CGs is sports injury prevention (franke Herzog) and that to our best knowledge there have been no RCTs on the effects of CGs on RRI recurrence or prevention in athletes who participate in sports that require any level of running.(28) We believe that our results are of importance in order to highlight this important gap of knowledge and show the potential of target trial emulations in sports medicine by attempting to apply it to the used of CGs. (Line 285-292)

**Comment 9**
My specific comments and suggestions for improvement are listed below:

1. The authors correctly followed the CONSORT guidelines by not performing significance testing (p-values) for baseline characteristics. This is methodologically sound, as any differences in a randomized setting (or its emulation) are due to chance. However, I recommend the authors briefly comment in the text whether any clinically relevant imbalances were observed between the groups (e.g., age, previous injury history, or weekly mileage) that could potentially act as confounders, despite the lack of formal statistical testing.

**Response 9**

We have added the following to the manuscript

Line 198-201 “In order to provide insight into the comparability of the group CG-users and non-CG-users standardized mean differences (SMD) between these groups were reported using the CG-users group as the reference. An SMD <.2 was considered as a small difference, <.5 was considered a moderate difference, <.8 a large difference. ([Cochrane handbook](https://www.cochrane.org/authors/handbooks-and-manuals/handbook/current/chapter-15#section-15-5), Cohen 1998)”

Line 237-241 “Based on the SMDs between the groups of CG-users and non-CG-users there were only small differences between these groups on gender, age, length, and training goal. A moderate between group difference was observed for weight and running experience. The group non-CG-users had a higher average weight and more often reported running experience than the group of CG-users.”

Line 243, Table 2 – we have added the Standardized Mean Difference between the group CG-users and non-CG-users to the table for all cohorts combined.

**Comment 10**
2. The current adjustment for confounding (limited to only three variables) appears excessively simplistic for a study framed as a Target Trial Emulation. The fundamental goal of this methodological framework is to statistically replicate the balance of a RCT in an observational setting where real randomization is absent. To achieve this, a much more comprehensive set of covariates is required to ensure that the groups are truly comparable. Specifically, the authors have omitted critical factors that directly influence both the risk of RRI and the likelihood of using CGs, such as weekly training mileage, body mass index, age, and concurrent injury prevention strategies (e.g., strength training). Without accounting for these key variables, the study is highly susceptible to residual confounding, which undermines the validity of the emulation. I strongly recommend that the authors either include these factors in their analysis or, at a minimum, acknowledge this as a major limitation that compromises the target trial approach.

**Response 10**

Unfortunately, the SUMMUM-2017 study did not collect data on external and internal training load and concurrent injury prevention strategies. We do have data on the BMI and age of the runners, however our sample size is not sufficient to include these covariates in the analysis. We therefore added the following text to the limitations section of the manuscript on line 349-356:
“Based on the limited sample size of our study we also chose not to include covariates such as body mass index and age for which the data was available. There also were covariates of interest for which there was no data within the SUMMUM-2017 study did contain data on important covariates such as internal (such as rated perceived exertion) and external training load (per example weekly training milage), CG pressure gradient, the use of concurrent prevention strategies (per example strength training). In order to optimize statistical power for the main analysis, we applied a random effect model that allowed us to combine all cohorts and all available data. As the study did not contain data on all potential confounders, there is a risk of residual confounding.”

**Comment 11**
3. The group of runners using CGs is significantly smaller than the non-user group, which, combined with the relatively short follow-up period, raises concerns regarding the study's statistical power. In cases where no significant difference is found, it is crucial to determine if the study was adequately powered to detect a clinically meaningful effect. I suggest that the authors perform a post-hoc power analysis (or better yet, a calculation of the minimum detectable effect size) based on the achieved n and the observed frequency of RRIs. This would help clarify whether the 'null' finding is a true lack of effect or simply a result of an underpowered sample. Without this information, the conclusion that CGs are not effective for RRI prevention remains speculative.

**Response 11**

We agree with the reviewer that our sample size is relatively low. However, we have not added a post-hoc power analysis as this is not advised in the literature, see for example [Heinsberg et al. 2023](https://pmc.ncbi.nlm.nih.gov/articles/PMC9452450/#:~:text=observed%20statistical%20results.-,We%20encourage%20authors%20and%20peer%2Dreviewers%20to%20avoid%20using%20or,they%20are%20simply%20not%20informative.), Zhang 2019, [Hoenig & Heisey (2001)](https://sciences.ucf.edu/biology/pascencio/wp-content/uploads/sites/24/2016/11/HoenigHeisey2001.pdf), [Levine & Ensom (2001)](https://europepmc.org/article/MED/11310512).

Levine & Ensom 2001 reported that it is preferred to use the 95% confidence interval (CI) in order to inform our readers regarding appropriateness of our sample size for the analysis performed. Within our limitations section we admit that the limitations of our sample size is reflected in the wide confidence intervals (line 343-345).

“So, the sample size of our study is limited, which is reflected in the wide confidence intervals, and we did not perform any sample size calculation as would have been performed for a RCT.”

**Comment 12**

4. A significant limitation of this study is the lack of granularity regarding the compression garments (CGs) used by the participants. The term compression garments encompasses a wide variety of products with different compression levels (measured in mmHg), varying sizes, and different designs (e.g., calf sleeves vs. full socks). Since the effectiveness of CGs is highly dependent on the pressure gradient and the specific mechanical support provided, the authors should discuss whether the lack of standardization in the type and dosage of compression could have masked a potential preventive effect. At the very least, this should be acknowledged in the Limitations section.

**Response 12**

Unfortunately, the SUMMUM-2017 study did not collect data on external and internal training load and concurrent injury prevention strategies. We do have data on the BMI and age of the runners, however our sample size is not sufficient to include these covariates in the analysis. We therefore added the following text to the limitations section of the manuscript on line 349-356:
“Based on the limited sample size of our study we also chose not to include covariates such as body mass index and age for which the data was available. There also were covariates of interest for which there was no data within the SUMMUM-2017 study did contain data on important covariates such as internal (such as rated perceived exertion) and external training load (per example weekly training milage), CG pressure gradient, the use of concurrent prevention strategies (per example strength training). In order to optimize statistical power for the main analysis, we applied a random effect model that allowed us to combine all cohorts and all available data. As the study did not contain data on all potential confounders, there is a risk of residual confounding.”

**Comment 13**
Minor comments:
Keywords: I suggest revising the keyword list to avoid repeating terms already present in the title (e.g., 'compression garments', 'prevention'). Keywords should ideally complement the title to enhance the article's discoverability in database searches. Please consider replacing redundant terms with broader or related technical descriptors.

**Response 13**

Thank you for this advice. We have now changed the keywords to running; running-related injuries; compression stockings; target trial emulation

**Comment 14**

Abstract: The phrasing 'who used using CGs' is grammatically incorrect. Please revise this sentence to improve clarity.

**Response 14**

We have removed “using”.

**Comment 15**

Please remove the 'n' in 'an RRI' throughout the text

**Response 15**

We have replaced ‘an RRI’ with ‘a RRI’ throughout the manuscript.
