# Supplementary material for: The effectiveness of compression garments on RRI prevention in half- and full-marathon runners: Emulation of a RCT using data from an observational study
Source: PLoS One. 2026 Nov 12;21(11):e0362483. doi: 10.1371/journal.pone.0362483 (PMC13759087; doi:10.1371/journal.pone.0362483)
Supplement: Supplementary file 3 [file pone.0362483.s004.docx]

**Detailed comment-response**

**Comment 1:**

Journal Requirements:

If the reviewer comments include a recommendation to cite specific previously published works, please review and evaluate these publications to determine whether they are relevant and should be cited. There is no requirement to cite these works unless the editor has indicated otherwise. 

**Response 1**

We will account for the abovementioned journal requirement.

**Comment 2:**

Reviewers' comments:

Reviewer's Responses to Questions

Comments to the Author

1. If the authors have adequately addressed your comments raised in a previous round of review and you feel that this manuscript is now acceptable for publication, you may indicate that here to bypass the “Comments to the Author” section, enter your conflict of interest statement in the “Confidential to Editor” section, and submit your "Accept" recommendation.

Reviewer #1: All comments have been addressed

Reviewer #2: All comments have been addressed

2. Is the manuscript technically sound, and do the data support the conclusions?

The manuscript must describe a technically sound piece of scientific research with data that supports the conclusions. Experiments must have been conducted rigorously, with appropriate controls, replication, and sample sizes. The conclusions must be drawn appropriately based on the data presented.

Reviewer #1: Yes

Reviewer #2: Yes

3. Has the statistical analysis been performed appropriately and rigorously?

Reviewer #1: N/A

Reviewer #2: Yes

4. Have the authors made all data underlying the findings in their manuscript fully available?

The [PLOS Data policy](http://track.editorialmanager.com/CL0/http:%2F%2Fwww.plosone.org%2Fstatic%2Fpolicies.action%23sharing/1/010f019f20db51d3-a980757d-0d89-4442-bb54-6e74230bc35e-000000/--k9wofoGeqZjtZ5yOUtRgsU4-QKz-8tajQngl_6kH0=258) requires authors to make all data underlying the findings described in their manuscript fully available without restriction, with rare exception (please refer to the Data Availability Statement in the manuscript PDF file). The data should be provided as part of the manuscript or its supporting information, or deposited to a public repository. For example, in addition to summary statistics, the data points behind means, medians and variance measures should be available. If there are restrictions on publicly sharing data—e.g. participant privacy or use of data from a third party—those must be specified.

Reviewer #1: Yes

Reviewer #2: Yes

5. Is the manuscript presented in an intelligible fashion and written in standard English?

PLOS ONE does not copyedit accepted manuscripts, so the language in submitted articles must be clear, correct, and unambiguous. Any typographical or grammatical errors should be corrected at revision, so please note any specific errors here.

Reviewer #1: Yes

Reviewer #2: Yes

**Response 2**

We thank the reviewers for recognizing the abovementioned characteristics of our manuscript.

**Comment 3**

6. Review Comments to the Author

Please use the space provided to explain your answers to the questions above. You may also include additional comments for the author, including concerns about dual publication, research ethics, or publication ethics. (Please upload your review as an attachment if it exceeds 20,000 characters)

Reviewer #1:

This revised manuscript addresses a clinically meaningful and under-studied question, namely whether the use of lower-leg compression garments is associated with a change in the risk of lower-extremity running-related injuries in half- and full-marathon runners. The authors do not run a new trial. Instead they re-use the prospective SUMMUM-2017 cohort and analyse it within a target trial emulation framework, which is an honest and methodologically current way of squeezing causal information out of observational data. The central result is a null one. After adjustment, the odds of any lower-extremity running-related injury in any two-week window were essentially unchanged for compression-garment users versus non-users. The reporting of the emulation steps in Table 1 is transparent, the limitations section is candid, and the revision has answered several of the first-round requests, including the addition of standardized mean differences and the intraclass correlation coefficient.

The principal weaknesses are not stylistic but quantitative. The primary effect estimate is reported with two different values in the same paper, the time-effect p-values in Table 4 do not correspond to their confidence intervals, and the cohort 3Mo sensitivity result is internally contradictory.

**Response 3**

We thank the reviewer for his/her thorough work and critical thinking. In Table 4 the results of the emulation are provided and the odds ratios for the time variable. We have corrected the p-values in Table 4 by re-extracting them from the results of the analysis. The correct results are now shown in Table 4.

Regarding the 3-month cohort result. Due to the relatively low sample size for the sensitivity analysis, it may be possible that the effect estimate was unprecise and therefore yielded what appeared to be internally inconsistent results. To emphasize this, we added the following text to the manuscript on line 357-359: “Further, for the sensitivity analyses the sample size in each cohort is relatively small, as is reflected by the wide 95% confidence intervals for the odds ratios. The significant effect at 3Mo was based on only 41 participants and may represent an accidental finding.”

**Comment 4**

Beyond these numerical problems, the intervention itself is essentially undefined because the dataset holds no information on garment pressure, type, or compliance, and the outcome relies entirely on self-report with no test-retest reliability quantified in this cohort. The introduction also contains several unreferenced and self-referential statements. None of these problems is fatal to a null observational study presented as a proof of concept, but the statistical inconsistencies must be resolved before the paper can be trusted by readers.

**Response 4**

We agree with the reviewer that it would have been more optimal if additional data, as mentioned above by the reviewer, on the CGs and their use were available. However, unfortunately this is not the case. The data on the CG’s and their use which is available to the authors has been used in this manuscript. In table 1 and on:

- Line 130-132: “Runners were also asked whether they use CGs. No data on the type of CG or compliance for using CGs were registered.”
- line 186-189 we describe the data we have “In the SUMMUM-2017 study the runners, preparing for the Utrecht (half)marathon, reported at baseline whether they use CGs while they are running. That indicates factual use at that moment and was viewed as an intention to use CGs during the whole follow-up period. However, both the type of CG and the actual use of CGs after baseline were not reported in the observational study performed.”
- Regarding the introduction and unreferenced statements, we have adapted the sentence on line 67-69 to ‘The CGs are primarily used by endurance athletes to prevent RRIs or to improve post-exercise recovery, performance, and recovery from RRIs, although prevention of RRIs is the most important reason given by runners for using CGs. (8)’

Furthermore, we have added a reference to the sentence on line 77-78 ‘To date, no randomized controlled trials (RCTs) have investigated the primary or secondary preventive effects of CGs. (15)’

**Comment 5**

Strengths
The research question is genuinely relevant. Compression garments are heavily marketed and widely worn for injury prevention, yet the evidence base for that specific claim is thin, so a well-reported null result has value for clinicians and runners.
The target trial emulation is set out clearly. The nine-component structure in Table 1, separating the observational study, the hypothetical trial, and the emulation, makes the assumptions inspectable and is a good template for the field.
The analytical model is appropriate in principle. A mixed logistic regression with a random intercept correctly handles the repeated two-week measurements within runners, and the revision now reports the intraclass correlation coefficient (0.56), which quantifies the clustering the model is accounting for.

The revision is responsive. The keyword redundancy, the standardized mean differences for baseline comparability, and the expanded limitations on training load and garment pressure all improve the paper. The authors are also correct, against the reviewers, that a post-hoc observed-power calculation would add nothing, and their citation of the Hoenig and Heisey argument is sound. I support that decision.

**Response 5**

We thank the reviewer for recognizing the strengths and genuine importance of our manuscript.

**Comment 6**

Critical issues
Contradictory primary effect estimate. The Abstract (page 35, line 38, single line spacing) and the plain-language summary (page 36, line 55) report the primary effect as odds ratio 1.33, 95% CI 0.52 to 3.39. The Results text (page 49, lines 263 to 265) and Table 4 (page 50) report odds ratio 1.07, 95% CI 0.48 to 2.40 for compression-garment use. These are not the same number and the paper never explains which is the primary estimate or how the two were derived. A reader cannot tell whether 1.33 is an unadjusted estimate, a typographical residue from an earlier model, or an error. This single inconsistency undermines the headline finding and must be corrected so that one clearly defined adjusted estimate appears identically in the Abstract, the summary box, the Results, and Table 4.

**Response 6**

We thank the reviewer for highlighting this mistake made by us. The 1.33 odds ratio was from a previous model and is therefore an error. We corrected the odds ratio on line 36 and line 53, to 1.07 (95% CI 0.48 – 2.40).

**Comment 7**

Misaligned p-values in Table 4.

The time-category rows in Table 4 (page 50) are internally inconsistent. For example, 14 weeks before the Utrecht Marathon shows odds ratio 0.21 (0.09 to 0.50) with p = 0.36, yet a confidence interval that excludes 1.00 cannot carry a p-value of 0.36. Conversely, 2 weeks before shows 0.65 (0.37 to 1.12), an interval that includes 1.00, paired with p = 0.0025. The p-value column appears shifted relative to the estimates. Every odds ratio, confidence interval, and p-value in this table must be re-extracted from the model output and re-checked for correspondence, because as printed the table is not believable.

**Response 7**

We have corrected the p-values in Table 4 by re-extracting them from the results of the analysis. The correct results are now shown in Table 4.

**Comment 8**

Self-contradictory sensitivity result.

In the Sensitivity analyses (page 49, lines 273 to 275) the cohort 3Mo result is given as odds ratio 0.07, 95% CI 0.006, p = 0.80, and described as a significant effect in favour of compression garments. A p-value of 0.80 is not significant, and the confidence interval is malformed because only one bound is printed. With only 41 runners in this cohort and very few users, this estimate is almost certainly an unstable artefact of sparse data. The claim of a significant protective effect should be removed, the confidence interval reported in full or the cohort flagged as non-estimable, and the instability acknowledged rather than interpreted.

**Response 8**

We have corrected the p-value on line 275-278. Further we have adjusted the paragraph to put less emphasis on the significance of the results: “We analyzed each cohort separately in a sensitivity analysis. Appendix 2 shows the odds ratios per cohort. The odds ratio for cohort 3Mo showed a significant effect in favor of CGs (OR 0.07, 95% CI 0.006 - 0.80, p=0.0325). For the cohorts 4Mo, 2Mo and 1Mo no significant findings were found.”

Furthermore, a sentence was added to the limitations section in which we admit that the small sample size is the probable cause of the significant finding as reflected. (line 357-359) “Further, for the sensitivity analyses the sample size in each cohort is relatively small, as is reflected by the wide 95% confidence intervals for the odds ratios. The significant effect at 3Mo was based on only 41 participants and may represent an accidental finding.”

**Comment 9**

The intervention is undefined.

Compression garment use is recorded only as a yes or no at baseline (Table 1, component 4; page 41). There is no garment type, no interface pressure in mmHg, no information on whether and how often the garment was actually worn during the sixteen-week follow-up, and no in vivo pressure measurement device was used at any point. The authors acknowledge this, but because the exposure is the whole point of the study, the manuscript should state explicitly and early (Methods, page 44) that the analysis tests intention to use a self-selected garment, not a defined dose of compression, and the Conclusion should be worded so that the null result is not read as evidence that compression itself is ineffective.

**Response 9**

To emphasize the statements made by the reviewer, we added the following text to the manuscript:

- Line 186-189: In the SUMMUM-2017 study the runners, preparing for the Utrecht (half)marathon, reported at baseline whether they use CGs while they are running. That indicates factual use at that moment and was viewed as an intention to use CGs during the whole follow-up period. However, both the type of CG and the use of CGs after baseline were not reported in the observational study performed.
- Line 339-344: “Unfortunately, as in our study, none of the other studies measured CG pressure or registered compliance with CG use during the follow-up period, making it impossible to study differences between full and partial CG use. Consequently, also in our study, a firm conclusion whether the self-selected CGs are effective to prevent RRIs is hard to make, and the null results cannot be interpreted as a fact that compression in itself would have no effect on the prevention of RRIs.”

Furthermore, we altered the conclusion to – line 417-420: “We studied the use of a self-selected CG in half and full marathon runners, who had no RRIs at baseline, on the occurrence of lower extremity RRIs during their preparation for the 2017 Utrecht Marathon running event. We identified no significant effects of CGs use on the occurrence of lower extremity RRIs in these runners.”

And in the conclusion section:

Line 419-422: We identified no significant effects of CGs on the occurrence of lower extremity RRIs in these runners. However, because no compression grade of compliance was registered in this study, it does not automatically mean that compression in itself would have no effect on prevention of RRIs.

And in Line 424-428: “Future studies, both observational studies and RCTs, on the incidence of (recurring) RRIs in both uninjured and injured half and full marathon runners are needed. By also investigating the effects of CGs while (periodically) measuring the in vivo pressure provided by the CGs, and compliance, these studies will further contribute to answer the question whether CGs can help to prevent and reduce RRIs.

**Comment 10**

Important additions required
Reliability of the outcome and exposure. As an original study the manuscript should report the test-retest reliability of its primary measurement. The Dutch OSTRC is cited for its minimally important change and smallest detectable change (reference 23), but no test-retest reliability statistic (ICC, standard error of measurement, or coefficient of variation) is given for the injury outcome in this marathon cohort, and the single-time-point self-report of garment use has no reliability check at all. Add the available reliability evidence in the Methods (Questionnaires, page 39) and state plainly that exposure reliability could not be assessed.

**Response 10**

We have looked into in the dataset of our whole SUMMUM 2017 study whether it is possible for us to estimate the test-retest reliability of the OSTRC as requested by the reviewer. If we were able to perform a reliability analysis we would investigate the test-retest reliability of the OSTRC using Cohen’s Kappa (the OSTRC sum score is an ordinal variable). Unfortunately, we do not have a sufficient sample of runners whose injury status was unchanged and measured on two consecutive occasions that are close in time. The measurement interval between to OSTRC-questionnaires was always two weeks, and there are not enough runners for whom we can be certain that their injury status is unchanged. We assessed the change in injury status based on the anchor questionnaires that were included in the SUMMUM-2017 study. So, we do not have the data to perform this reliability analysis. However, in the international literature we identified 3 manuscripts reporting on the reliability of the OSTRC ([Kaewkul et al 2021](https://openurl.ebsco.com/EPDB%3Agcd%3A11%3A37468171/detailv2?sid=ebsco%3Aplink%3Ascholar&id=ebsco%3Agcd%3A148276149&crl=c&link_origin=www.google.com),  [Antonopoulos et al. 2025](https://journals.sagepub.com/doi/10.1177/17479541251333938), [Alimoradi et al 2026](https://pmc.ncbi.nlm.nih.gov/articles/PMC13088847/)) We included these as references. Therefore, we added the following text to the manuscript: line 373-381: “Further, we were unable to estimate the test-retest reliability of the Dutch version of the OSTRC questionnaire used because there were insufficient runners whose injury status was unchanged in two consecutive measurements. Nonetheless, test-retest reliability of the OSTRC questionnaire on health problems in other languages is reported in three studies. For the Thai version (first version of the OSTRC on health problems) a test-retest reliability intraclass correlation coefficient (ICC) of 0.970, 95% CI 0.950 to 0.982 in 57 thai athletes was reported. (45) For the Greek version of the second version of the OSTRC a test-retest reliability ICC of 0.93, 95% CI 0.89–0.97, in 56 female volleyball athletes was found. (46) And for the Persian version (OSTRC-second version) reported a test-retest reliability ICC range: 0.82–1.00 in 207 elite Persian athletes from various sports. (47)”

**Comment 11**

Precision rather than power.

The decision not to compute observed power is correct, but the manuscript should replace the vague appeal to wide confidence intervals with the minimum detectable effect, that is, the smallest odds ratio the achieved sample of 70 users among 329 runners could have detected at alpha 0.05. Reporting this critical effect size (Data analysis, page 44) converts a null finding into an informative statement about what the study could and could not have ruled out.

**Response 11**

On suggestion of the reviewer we added the following text to line 352-354 “Alternatively, we calculated a minimal detectable effect of approximately 0.36. This is based on the incidence of injury in non-CG users, the total number participants in the SUMMUM-2017 study and the proportion of these participants using a CG and the ICC as reported in our study.”

**Comment 12**

Events per variable.

Reviewer 1 asked for the number of injury events per predictor and this was not added. With four confounders plus seven time categories in the model, state the events-per-variable so readers can judge model stability (Data analysis, page 44).

**Response 12**

In total, we observed 172 injuries and estimated 10 ORs in the primary analyses, leading to an events per variable ratio of 17.2. The events per variable ratio, however, was reported as a measure of model stability for ordinary logistic regression. Events per variable as a rule was criticized (see [Van Smeden et al. BMC Medical Research Methodology (2016) 16:163](https://pubmed.ncbi.nlm.nih.gov/27881078/)), as somewhat of a simplification. Furthermore, we are unaware of any reference that allows us to generalize this to mixed logistic regression models as used in this study. We are therefore very reluctant to report this as a measure of model stability is, as we are unsure of the validity of this measure.

**Comment 13**

A figure with error bars.

The only place in this manuscript where error bars are meaningful is the presentation of the odds ratios. A forest plot of the compression-garment estimate and the confounders, with the 95% confidence intervals drawn as horizontal error bars around a vertical line at 1.00, would communicate the precision of the null far better than Table 4 alone. I recommend adding it as a new figure beside Table 4 (page 50).

**Response 13**

We agree that forests plots with error bars are informative figures. However, in the case of this manuscript we chose not to use a figure but rather present a table with the odds ratio’s because of the multivariate analysis performed.

**Comment 14**

Pre-specification of the emulation.

This is not a systematic review, so neither PROSPERO registration nor the systematic-review reporting primer applies, and I do not ask for them. However, target trial emulations are strengthened by a pre-specified protocol and analysis plan. State in the Methods (page 44) whether the emulation protocol and confounder set were defined before analysis or chosen afterwards, since data-driven confounder selection would change how the result should be read.

**Response 14**

As requested by the reviewer we have added the following statement to the method section, specifically the data analysis paragraph on line 214-216 “. These potential confounders were specified prior to the analyses.” And on line 170-171: “The emulation protocol was defined before the analyses.”

**Comment 15**

Minor corrections
Introduction, unreferenced and self-referential sentences. Every claim in the Introduction should carry a citation. Two sentences currently do not. The statement that a variety of measures have been used to prevent, mitigate, or cure running-related injuries (page 37, lines 66 to 67) needs a reference. The statement that garments are also used to improve post-exercise recovery, performance, and recovery from injuries, together with the claim that prevention is the main reason athletes give (page 37, lines 70 to 73), is currently asserted without support and reads as self-referential; attach references and repair the run-on sentence.

**Response 15**

We have added a reference on line 65, after the sentence on a variety of measures have been used to prevent, mitigate, or cure running-related injuries. Additionally, I would like to inform the reviewer that indeed some of the references are ‘self-referential’. The reason is that these manuscripts are part of the PhD thesis of the first author Thierry Franke. The manuscripts build upon one another.

Furthermore, we have added a reference on line 67-69 after the sentence that states that prevention is the main reason for CG use. “The CGs are primarily used by endurance athletes to prevent RRIs or to improve post-exercise recovery, performance, and recovery from RRIs, although prevention of RRIs is the most important reason given by runners for using CGs. (8)”

**Comment 16**

Repeated typographical errors. Target trail should be target trial throughout Table 1 and the Discussion (for example page 54, lines 389 to 391). Post-exercise recover should be recovery (page 38, line 77). The used of CGs should be the use of CGs (page 51, line 292). Angelmeyer and Anglemyer are both used for the same author within two lines (page 52, lines 325 and 327); use one consistent spelling. The Table 4 title odds ratio's should be odds ratios (page 50).
Hyphenation. 12months should be 12 months or 12-months consistently (page 46, lines 234 to 235).
Reference list formatting. Entry 31 (Moen, M.H., Holtslag, L., Bakker E ...) uses an author format different from the rest of the list; standardize it to the vancouver style used elsewhere.

**Response 16**

We thank the reviewer for pointing out the typographical errors. The following alterations were made;

- Lines 388, 392 and table 1 ‘trail’ changed into ‘trial’
- Line 75 ‘recover’ 🡪 ‘recovery’
- Line 294 ‘used of CGs’ 🡪 ‘use of CGs’
- Line 329 ‘Angelmeyer’ 🡪 ‘Anglemeyer’
- Table 4 ‘odds ratio’s’🡪 ‘odds ratios’
- Line 237 12months 🡪 12-months
- Reference entry 31 has been updated.

**Comment 17**

Decimal, formatting and device verification
The decimal separator is the period throughout, which is correct. Three issues remain. First, the standardized mean difference thresholds are written as less than .2, less than .5, and less than .8 (Data analysis, page 44, lines 200 to 201); these should carry a leading zero and two decimals, as 0.20, 0.50, and 0.80. Second, confidence intervals are punctuated inconsistently: the Abstract uses an en dash (0.52 to 3.39 written with a dash), while Table 4 uses a semicolon (0.48; 2.40). Choose one convention and apply it everywhere; the clearest is to separate the bounds with the word to, for example 0.48 to 2.40. Third, p-values are reported to a variable number of decimals (0.86 in one row, 0.0005 in another); report them to a consistent precision, giving exact values rather than threshold labels, which the authors already mostly do.

**Response 17**

The following changes were made:

Line 2020-203: ‘An SMD <0.20 was considered as a small difference, 0<.50 was considered a moderate difference, <0.80 a large difference.(26)’

Table 4: 95%CI are now written with a dash (e.g. (0.48 - 2.40)).

**Comment 18**

On instrumentation, the study is questionnaire-based, so there is no biomechanical sensor with a sampling frequency to report. The measurement instrument, the Dutch OSTRC, is named with a validity reference, and its administration interval (every two weeks) is stated. The delivery platform is identified with manufacturer and country (NetQuestionnaires, NETQ Healthcare BV and NETQ Insights BV, Utrecht, The Netherlands) but without a validation reference; note this. The statistical software is fully specified (SAS v9.2, SAS Institute Inc., Cary, NC, USA). The one device that is entirely unspecified is the compression garment itself, which has no brand, model, country, interface pressure, or validation reference, and no pressure monitor was used to verify the dose delivered. This is the most important instrumentation gap and should be stated as such.

**Response 18**

Indeed, the reviewer sharply noticed the measurement instruments and software used. Additionally, also he/she restates the previously mentioned shortcoming of our study, that we have little knowledge of the CGs used. We have continuously been candid regarding this shortcoming of our study. Additionally, in this second revision – based on the comments from the reviewers we have added:

On line 97-98 “Within the target trial emulation we aim to analyze the use of a self-selected CG.”

On line 130-132 “. Runners were also asked whether they use CGs. No data on the type of self-selected CG or compliance for using CGs were registered”

On line 417-418 “We studied to use of a self-selected CG in half and full marathon runners, who had no RRIs at baseline,…”

**Comment 19**
Suggested references and where to place them
The following seven references are suggested to strengthen specific passages. They are listed here with the rationale and exact location; full bibliographic details follow at the end of this report. The choice reflects topical relevance rather than sequence.
1. Introduction, page 37, lines 66 to 67, double spacing, at the sentence on measures used to prevent running-related injuries. Cite Naderifar et al. (2024), which shows that neuromuscular control training reduces ankle sprains, to support the claim that preventive measures exist and have been tested, removing the current self-referential phrasing.
2. Methods, Data analysis, page 44, lines 202 to 208, at the description of the multivariable logistic model. Cite Costa et al. (2023), an epidemiological study of foot and ankle injuries analysed by multivariate logistic regression, as a methodological precedent for modelling lower-extremity injury risk with adjusted odds ratios.
3. Discussion, page 52, lines 321 to 324, where the growing popularity of target trial emulation and real-world data is discussed. Cite Ng et al. (2023) on the future and virtualization of clinical trials, to place the emulation approach within the broader move toward real-world and virtual trial designs.
4. Discussion, limitations, page 53, lines 366 to 367, at the recommendation to capture external training load with GPS data. Cite Padulo et al. (2019), which establishes the validity and reliability of a low-end 50 Hz GNSS receiver during running, providing a validated tool for the very measurement the authors recommend for future work.
5. Discussion, limitations, page 53, lines 366 to 368, at the recommendation to capture internal training load with rated perceived exertion. Cite Padulo et al. (2014), on the construct validity of session-RPE, to support session-RPE as a validated internal-load measure for future running studies.
6. Discussion, biomechanical mechanism paragraph, page 52, lines 317 to 320 (and linked to the under-referenced Introduction sentence on mechanisms). Cite Padulo et al. (2025), on gait variability and fatigability during a simulated 10 km race, to give a concrete fatigue-related mechanism by which lower-leg interventions might plausibly influence injury risk in distance runners.
7. Methods and future-research framing, supporting the reliability point and the Conclusion call for better measurement (page 54, lines 402 to 406). Cite Padulo et al. (2026), which determines the minimum number of steps needed for reliable kinetic, kinematic, gait-variability, and oxygen-uptake parameters in outdoor running, to underpin the recommendation that future studies use reliable running-gait measurement.

**Response 19**

We have added another reference than suggested on the method section related to the description of the multivariable logistic model, namely: Fitzmaurice Laird & Ware, Applied longitudinal analyses, Wiley, 2004 on line 205.

Further we have added:

- Ng et al. (2023) on line 324-326 “Target trial emulation methodology is becoming increasingly popular to assess the relative effectiveness of interventions and can be placed in a broader development wherein trials can be performed both in the real-world and virtual.”
- Padulo et al (2014) on line 362
- Padulo et al. (2014 and 2019) on line 388

**Comment 20**

Overall recommendation
Major revision. The study design, the analytical model, and the honesty of the limitations are acceptable for a null observational proof of concept, and several first-round comments have been addressed well. However, the manuscript cannot proceed while the primary odds ratio is reported with two different values, the Table 4 p-values do not match their confidence intervals, and the cohort 3Mo sensitivity result is internally contradictory. Once these numerical inconsistencies are corrected, the reliability and precision additions are made, the intervention is explicitly framed as an undefined self-selected exposure, and the Introduction is fully referenced, the paper would make a useful and appropriately modest contribution.

Suggested references (full details)
1. Naderifar H, Babakhanian S, Najafi-Vosough R, Karimizadeh Ardakani M. Neuromuscular Control Training is Effective to Prevent Ankle Sprains in Athletes. Muscles Ligaments Tendons J. 2024;14(1):188-196. doi:10.32098/mltj.01.2024.16.
2. Costa LPB, Correa FG, Cunha HCM, Maciel ES, Lemos AVKC, Nery CAS, et al. Epidemiological Study of Foot and Ankle Injuries in Athletes: A Multivariate Logistic Regression Analysis. Muscles Ligaments Tendons J. 2023;13(3):430-435. doi:10.32098/mltj.03.2023.11.
3. Ng CE, Bowman S, Ling J, Bagshaw R, Birt A, Yiannakou Y. The future of clinical trials - is it virtual? Br Med Bull. 2023;148(1):42-57.
4. Padulo J, Iuliano E, Brisola G, Dello Iacono A, Zagatto AM, Lupo C, Fuglsang T, Ardigo LP, Cular D. Validity and reliability of a standalone low-end 50-Hz GNSS receiver during running. Biol Sport. 2019;36(1):75-80. doi:10.5114/biolsport.2019.79974.
5. Padulo J, Chaabene H, Tabben M, Haddad M, Gevat C, Vando S, Maurino L, Chaouachi A, Chamari K. The construct validity of session RPE during an intensive camp in young male Karate athletes. Muscles Ligaments Tendons J. 2014;4(2):121-126. doi:10.11138/mltj/2014.4.2.121.
6. Padulo J, Borrelli M, Antiglio A, Esposito F. Gait variability and fatigability during a simulated 10-km running race in trained runners. Eur J Appl Physiol. 2025;125(9):2529-2535. doi:10.1007/s00421-025-05780-8.
7. Padulo J, Scurati R, Baldo S, Esposito F, Peyre-Tartaruga LA. Determining the minimum number of steps required for reliable kinetic, kinematic, gait variability, and VO2 parameters in outdoor running. Br Med Bull. 2026;157(1):ldaf025. doi:10.1093/bmb/ldaf025.

**Response 20**

We thank the reviewer for their extensive comments which helped us to further improve our manuscript. We have made changes to the manuscript (see other comments and responses), which address the summarized feedback points in comment 20. We think that therefore we have addressed the reviewers’ concerns and performed the major revision of our manuscript as requested. In response 19 we have stated which references we have added to the manuscript based on the addition of the editor of PlosOne that we are free to choose which references to add or not.
